# Violence against healthcare in conflict: a systematic review of the literature and agenda for future research

**DOI:** 10.1186/s13031-021-00372-7

**Published:** 2021-05-07

**Authors:** Rohini J. Haar, Róisín Read, Larissa Fast, Karl Blanchet, Stephanie Rinaldi, Bertrand Taithe, Christina Wille, Leonard S. Rubenstein

**Affiliations:** 1 Division of Epidemiology, University of California, Berkeley, School of Public Health, Berkeley, CA USA; 2 University of Manchester, School of Arts, Languages and Cultures, Humanitarian and Conflict Response Institute, Manchester, UK; 3 Geneva Centre of Humanitarian Studies, University of Geneva, The Graduate Institute of International and Development Studies, Geneva, Switzerland; 4 Insecurity Insight, Geneva, Switzerland; 5 Program on Human Rights, Health and Conflict, Center for Public Health and Human Rights, Bloomberg School of Public Health, Johns Hopkins University, Baltimore, MD USA

**Keywords:** Attacks on health, International humanitarian law, Medicine, Violence against healthcare, Hospitals, Conflict, War, Armed conflict, Geneva conventions, Protection, War crimes

## Abstract

**Background:**

Attacks on health care in armed conflict, including those on health workers, facilities, patients and transports, represent serious violations of human rights and international humanitarian law. Information about these incidents and their characteristics are available in myriad forms: as published research or commentary, investigative reports, and within online data collection initiatives. We review the research on attacks on health to understand what data they rely on, what subjects they cover and what gaps exist in order to develop a research agenda going forward.

**Methods and findings:**

This study utilizes a systematic review of peer-reviewed to identify and understand relevant data about attacks on health in situations of conflict. We identified 1479 papers published before January 1, 2020 using systematic and hand-searching and chose 45 articles for review that matched our inclusion criteria. We extracted data on geographical and conflict foci, methodology, objectives and major themes. Among the included articles, 26 focused on assessment of evidence of attacks, 15 on analyzing their impacts, three on the legal and human rights principles and one on the methods of documentation. We analyzed article data to answer questions about where and when attacks occur and are investigated, what types of attacks occur, who is perpetrating them, and how and why they are studied. We synthesized cross-cutting themes on the impacts of these attacks, mitigation efforts, and gaps in existing data.

**Conclusion:**

Recognizing limitations in the review, we find there have been comparatively few studies over the past four decades but the literature is growing. To deepen the discussions of the scope of attacks and to enable cross-context comparisons, documentation of attacks on health must be enhanced to make the data more consistent, more thorough, more accessible, include diverse perspectives, and clarify taxonomy. As the research on attacks on health expands, practical questions on how the data is utilized for advocacy, protection and accountability must be prioritized.

**Supplementary Information:**

The online version contains supplementary material available at 10.1186/s13031-021-00372-7.

## Introduction

Attacks on healthcare in armed conflict violate central tenets of human rights and International Humanitarian Law (IHL) [1]. These attacks, comprising physical violence as well as threats, intimidation and interferences with healthcare, are a frequent but underreported facet of international and intra-national armed conflicts. Health facilities such as health centers, hospitals and private medical offices, and transports such as ambulances and supply trucks, have been bombed, looted, burned, blocked, or occupied across various contexts over decades. Healthcare personnel and patients have been physically assaulted as well as arrested and jailed, intimidated, threatened, or blocked from receiving or providing care. Resolutions from the World Health Assembly, the United Nations (UN) Security Council, and the UN General Assembly have reiterated the critical need to protect health during conflict and the need for the World Health Organization (WHO) to compile data on these violations [2–5]. Interest in rigorous and systematic documentation and reporting on these attacks is growing from the public health, medical and legal sectors. Better documentation is central to understanding the true scope of the attacks on healthcare across contexts, exploring their burden on health systems and populations already impacted by conflict and violence, and could assist in developing stronger protection, advocacy and accountability mechanisms.

Increased advocacy and attention in recent years has prompted calls to scale efforts to compile data on the nature and extent of attacks on healthcare and their sequalae. Non-governmental organizations and the ICRC and WHO in particular have met this call with efforts to improve documentation of incidents of attacks on health and the dissemination of research and investigations [6, 7]. However, there has been significant variation in the objectives, types and geographic contexts of attacks as well as in the methods of data collection and the operational definitions used to investigate and describe them. Discrepancies in these elements may obscure important dynamics, such as why attacks occur and how they are counted, and limit comparison of datasets.

Interest in conflict data of all kinds and global programs to identify and document violence against civilians are growing alongside region-specific databases [8–11]. Initiatives focus on violence against civilians, airstrikes, terrorism events, security of humanitarian organizations and other facets of conflict [12–14]. The growth in conflict data initiatives has allowed quantitative, and increasingly disaggregated, analysis of conflict and conflict-related phenomena, albeit with limitations [15–21]. Within this broader conflict and human rights data landscape, research to document attacks on health and characterize their features can enable more timely and systematic monitoring of attacks.

Better understandings of attacks on healthcare can contribute to preventing attacks, mitigating their effects, bearing witness to the costs, and prosecuting the violations of IHL and international human rights law (IHRL) that they represent. Documentation of attacks can contribute to preventing attacks by identifying vulnerabilities, shaming perpetrators, and developing security strategies. Knowledge of the scope, scale and impact of attacks on health can help humanitarian actors target resources and programs towards those have been attacked and support recovery processes. The voices of survivors are powerful in condemning violence and deepening understanding of the social, psychological, physical and economic repercussions of attacks. Bearing witness and securing accountability for perpetrators and by exposing these attacks and their human toll is an important part of ensuring justice has been served.

There have already been several efforts to review some aspects of data on attacks on health in conflict. These include reviews focused on the impacts of attacks [22], reviews on the scope of attacks resulting from the Arab Spring, especially in Syria [23, 24], and on incidents involving health workers [25]. One rapid review covering 2011 to 2017 criticized aspects of the documentation process and the limitations of standardization in the field [26]. The present study employs a systematic review methodology to build upon these inquiries. As part of the ongoing Researching the Impact of Attacks on Healthcare project, we ask: what is the state of evidence about attacks on health in conflict across the decades [27]? What research has been carried out? For what purpose? Using what methodologies? And what still remains to be done? We aim to provide insight into the current state of evidence about attacks on healthcare and identify next steps for data collection, data utilization and research.

## Methodology

We conducted a systematic review of articles and reports that document attacks or analyze related risks, methods and/or impacts.

### Data sources and search strategy

We utilized a methodology based on the Preferred Reporting Items for Systematic Reviews and Meta-Analysis (PRISMA) guidelines to identify peer-reviewed articles and reports pertinent to data collection and analysis around attacks on health in conflict settings. We reviewed multiple electronic databases for appropriateness and selected PubMed/Medline as it covered a broad range of topics on attacks on healthcare in armed conflict without overwhelming the search with irrelevant material. After empirically assessing relevant keywords, we identified pertinent articles that included the following terminology categories: (1) conflict or war, (2) attack (terms such as violence, bombing, arrest or torture) and (3) targeting health (including terms for facilities, transports, healthcare workers or patients). The full search strategy and MeSH terms can be found in Table 1S (supplementary). We reviewed titles and abstracts of all papers retrieved from the systematic search to capture relevant articles, and then reviewed the full text of potentially suitable articles prior to inclusion. Aware that many articles and reports relevant to the research question are not available in traditional biomedical article searches, we rigorously reviewed article references and searched grey literature by browsing published reports by international organizations involved in the provision of healthcare and those that reported on human rights or humanitarian law violations in relation to health care. We also consulted professional networks to identify additional papers that fit the inclusion criteria.

### Study selection

We framed a priori definitions of attacks, healthcare and conflict in the exploratory phase but remained open for additional input from the review. Initially, attacks were defined as violence, threatened or actual, as well as intimidation and interference with normal health functions and/or misuse or misrepresentation of the protected status of healthcare. We included non-physical and indirect violence in our definition to ensure we looked at these often overlooked but frequent types of incidents that nonetheless have serious impacts on the health system [28]. This broad definition of violence allows for a richer review of papers and aligns with the normative definitions of attacks on health by the WHO, ICRC and the Safeguarding Health in Conflict Coalition. We defined healthcare to include a diverse range of health services. Based on previous research, we included the domains of facilities, transports, patients (the wounded and sick), personnel and the protected status of healthcare as targets of attack [29]. The definition of conflict, for this study, was inclusive of the IHL definitions of international armed conflicts and non-international armed conflicts as well as other contexts that the authors of the article or the research team identified or referred to as conflict related [30, 31].

To ensure we covered both historical and current trends and conflicts, we conducted an open search for documents published before January 1, 2020, but we limited articles to English based on the competencies of the research team. We did not formalize a start date in order to be inclusive of older reports but we did not identify any articles published prior to 1983.

We excluded articles that focused broadly on humanitarian settings without mentioning conflict, as their findings may fall outside the remit of IHL or may be more focused on interpersonal violence rather than conflict-related violence. Using the search terms defined earlier, we collected relevant articles, removed all duplicates and selected a final list for in-depth analysis.

The selection process was designed to identify articles with a robust research on violence against healthcare in the context of conflict research. Any article deemed by both reviewers to not substantively (1) conduct formal research or deep analysis of attacks (violence/interference or threats) and (2) focus on healthcare (broadly defined) (3) in the context of armed conflict (as defined by the article) was excluded during the title and abstract review.

### Analysis

For the included articles, the reviewers extracted descriptive data such as timeframe, context and region, source data and its availability, type of study or program, conceptual framing of the text, methods and outcome measures. We used a deductive approach to extract type(s) of attacks studied, geographical focus of the article or program, methods/study design, limitations and findings. Then, we used an inductive approach to identify additional categories emerging from the articles and to further dissect the concepts. The themes included: primary focus (armed conflict or attack on health or other), objective of paper, definitions of conflict and attacks on healthcare and take-aways. The process of extracting these themes was iterative, requiring continual comparison across papers.

## Results

**Fig. 1:**
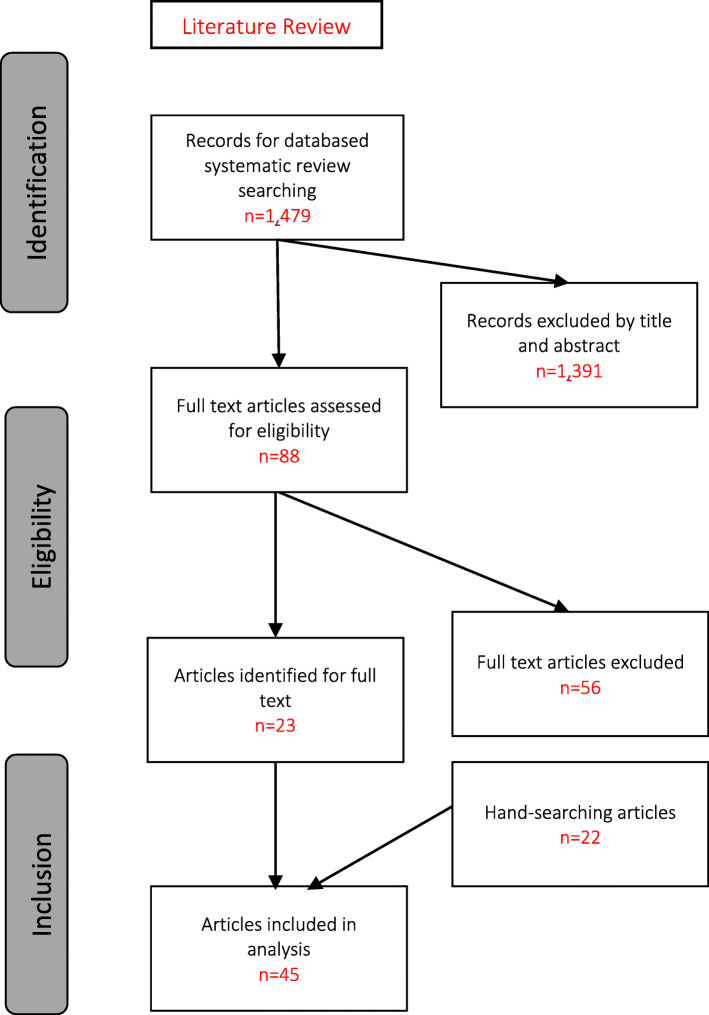
Search Strategy and Selection

We identified 45 articles for in-depth review. Our systematic search yielded 1479 discrete articles, of which we selected 23 articles (Fig. 1). An additional 22 articles were identified though references review and professional networks. The full list of papers appears in Table 1: Study Summaries [22–24, 26, 29, 32–87].

**Table 1:** Summaries of the 45 articles included in the review

Type	Citation	Geographical context	Thematic domain	Study Design or Approach
Research	Briody C. 2018. Review of Attacks on Health Care Facilities in Six Conflicts of the Past Three Decades	Global	Documentation of Attacks	Retrospective open source data analysis
	Buckley CJ. 2019. An assessment of attributing public healthcare infrastructure damage in the Donbas five years after Euromaidan: implications for Ukrainian state legitimacy	Ukraine	Documentation of Attacks	Retrospective open source data analysis
	Burnham GM. 2009. Doctors leaving 12 tertiary hospitals in Iraq, 2004–2007.	Iraq	Impact of Attacks	Retrospective registry analysis
	Burnham G. 2012. Understanding the impact of conflict on health services in Iraq: information from 401 Iraqi refugee doctors in Jordan: Conflict and health services in Iraq	Iraq	Impact of Attacks	Qualitative interviews: Semi-structured approach
	Chi, PC. 2015. Perceptions of the effects of armed conflict on maternal and reproductive health services and outcomes in Burundi and Northern Uganda: a qualitative study	Burundi and Northern Uganda	Impact of Attacks	Qualitative interviews
	Chukwuma A. 2019. Armed conflict and maternal health care utilization: Evidence from the Boko Haram Insurgency in Nigeria	Nigeria	Impact of Attacks	Retrospective registry analysis
	Cliff J. 1988. Health as a target: South Africa’s Destabilization of Mozambique	Mozambique	Impact of Attacks	Retrospective registry analysis
	Elamein M. 2017. Attacks against health care in Syria, 2015–16: results from a real-time reporting tool.	Syria	Documentation of Attacks	Retrospective registry analysis
	Fardousi N. 2019 Healthcare under siege: a qualitative study of health-worker responses to targeting and besiegement in Syria	Syria	Documentation of Attacks	Qualitative interviews: Semi-structured approach
	Foghammar L. 2016 Challenges in researching violence affecting health service delivery in complex security environments	Global	Documentation of Attacks	Qualitative interviews and workshop discussions- Secondary analysis
	Footer KHA. 2014. On the frontline of eastern Burma’s chronic conflict - Listening to the voices of local health workers.	Myanmar	Documentation of Attacks	Qualitative interviews: Semi-structured approach
	Footer KHA. 2018. Qualitative accounts from Syrian health professionals regarding violations of the right to health, including use of chemical weapons, in opposition-held Syria	Syria	Documentation of Attacks	Qualitative interviews: Semi-structured approach
	Haar RJ. 2014. Measurement of attacks and interferences with health care in conflict: validation of an incident reporting tool for attacks on and interferences with health care in eastern Burma	Myanmar	Documentation of Attacks	Qualitative interviews: Semi-structured approach
	Haar RJ. 2018. Determining the scope of attacks on health in four governorates of Syria in 2016: Results of a field surveillance program	Syria	Documentation of Attacks	Retrospective registry analysis
	Hemat H. 2017. Before the Bombing: High Burden of Traumatic Injuries in Kunduz Trauma Center, Kunduz, Afghanistan	Afghanistan	Impact of Attacks	Retrospective registry analysis
	Lafta RK. 2019. Violence against health-care workers in a conflict affected city	Iraq	Documentation of Attacks	Retrospective cohort survey study
	Michlig GJ. 2019. Providing healthcare under ISIS: A qualitative analysis of healthcare worker experiences in Mosul, Iraq between June 2014 and June 2017	Iraq	Impact of Attacks	Qualitative interviews: Semi-structured approach
	Namakula J. 2014. Living through conflict and post-conflict: experiences of health workers in northern Uganda and lessons for people-centered health systems	Uganda	Impact of Attacks	Qualitative interviews study - life history approach
	Neuman M. 2014. “No patients, no problems:” Exposure to risk of medical personnel working in MSF projects in Yemen’s governorate of Amran	Yemen	Documentation of Attacks	Qualitative interviews
	RI S. 2019. Attacks on health facilities as an indicator of violence against civilians in Syria: An exploratory analysis of open-source data.	Syria	Documentation of Attacks	Retrospective open source data analysis
	Rytter MJH. 2006. Effects of armed conflict on access to emergency health care in Palestinian West Bank: systematic collection of data in emergency departments	Palestine	Impact of Attacks	Retrospective cohort survey study and registry analysis
	Sousa c. 2011. Conflict, health care and professional perseverance: A qualitative study in the West Bank	Palestine	Impact of Attacks	Qualitative interviews and Participant observation
	Sinha S. 2012. Vulnerabilities of Local Healthcare Providers in Complex Emergencies: Findings from the Manipur Micro-level Insurgency Database 2008–2009	India	Documentation of Attacks	Retrospective registry analysis
	Trelles M. 2016. Averted health burden over 4 years at Médecins Sans Frontières (MSF) Trauma Centre in Kunduz, Afghanistan, prior to its closure in 2015.	Afghanistan	Impact of Attacks	Retrospective registry analysis
	Ud Din I. 2012. “How the Taliban undermined community healthcare in Swat, Pakistan.”	Pakistan	Documentation of Attacks	Qualitative interviews: Semi-structured approach
	Wong CH. 2018. Ambulances under siege in Syria	Syria	Documentation of Attacks	Retrospective open source data analysis
	Zimmerman HL. 2019 Attacks on health in conflict: generating attention in the modern information landscape	Global	Documentation of Attacks	Qualitative interviews and focus group discussions and systematic literature review
Human Rights Investigation	Crombé X. 2019. War Breaks Out: Interpreting Violence on Healthcare in the Early Stage of the South Sudanese Civil War	South Sudan	Documentation of Attacks	Mixed methods human rights investigation
	Garfield RM. 1987. Health-Related Outcomes of War in Nicaragua	Nicaragua	Impact of Attacks	Mixed methods: Retrospective registry analysis and qualitative interviews
	Geiger J. 1989. A new medical mission to El Salvador.	El Salvador	Documentation of Attacks	Mixed methods human rights investigation
	Gellhorn A. 1983. Medical mission report on El Salvador	El Salvador	Documentation of Attacks	Mixed methods human rights investigation
	Eisenberg C. 1983. Health and human rights in El Salvador	El Salvador	Impact Analysis	Mixed methods human rights investigation
	Marton R. 2011. Human rights violations during Israel’s attack on the Gaza Strip: 27 December 2008 to 19 January 2009.	Palestine	Documentation of Attacks	Mixed methods human rights investigation
Policy Analysis / Legal	Bouchet-Saulnier F. 2018. An Environment Conducive to mistakes: lessons learned in Kunduz	Afghanistan	Law and Human Rights	In depth case study
	Foghammar L. 2014. Violence against healthcare in fragile systems	Global	Documentation	Incident review and policy analysis
	Footer KHA. 2013. Human Rights Approach to Healthcare in Conflict	Global	Law and Human Rights	Legal or policy analysis
	Gates S. 2017. Patterns of Attacks on Medical Personnel and Facilities: SDG 3 meets SDG 16	Global	Impact of Attacks	Legal or policy analysis
	Mclean D. 2019. Medical care in armed conflict: Perpetrator discourse in historical perspective	Global	Impact of Attacks	Historical case study analysis
	Terry F. 2013. Violence against health care: insights from Afghanistan, Somalia, and the Democratic Republic of the Congo	Afghanistan, DRC and Somalia	Methods description	Incident review and policy analysis
Reviews	Afzal MH. 2019. A scoping review of the wider and long-term impacts of attacks on health in conflict zones	Global	Impact of Attacks	Scoping review
	Boi-Karroum L. 2018. Health Care Workers in the setting of the “Arab Spring”: a scoping review for the Lancet-AUB Commission on Syria	MENA	Documentation	Scoping review
	Fouad FM. 2017. Health workers and the weaponisation of health care in Syria: a preliminary inquiry for The Lancet-American University of Beirut Commission on Syria	Syria	Documentation	Mixed methods: Literature review and qualitative stakeholder interviews
	Redwood-Campbell LJ. 2014. Health care workers in danger zones: a special report on safety and security in a changing environment	Global	Documentation	Qualitative review
	Rubenstein LS. 2010. Responsibility for protection of medical workers and facilities in armed conflict	Global	Documentation	Qualitative review

### Descriptive analysis of the literature

#### Objectives, focus and type of analysis in the literature

Of the 45 included articles, 35 (78%) focused exclusively on attacks on healthcare while nine concentrated on armed conflict but incorporated substantial discussions of attacks on healthcare. The objectives of the papers were broadly categorized into four domains: 26 papers reviewed incidents of attacks and their features or the immediate aftermath; 15 papers explored the impact of the attacks: on the health system; on health workers; or on the population. Three papers addressed the legal and human rights facets and implications of attacks on health and one paper described methods for documenting attacks.

In evaluating the approaches to the subject matter, 28 papers (60%) broadly fell under research, six papers were best described as human rights investigations (informal methods that included visual inspection, interviews, forensics and/or document review), five papers were scoping or literature reviews, and another six papers analyzed law and/or policy. Among the 28 research articles, 15 reported primarily on quantitative analysis of incidents of attacks. Eight studies conducted secondary or retrospective data analysis using registry data from a hospital, health system, region, or country to document attacks (*n* = 3) or to analyze their impacts (*n* = 5). Three conducted retrospective cohort studies and four utilized publicly available data for secondary analysis. Thirteen articles were based on qualitative interviews, workshops or focus group discussions or a combination of these. While we did not formally assess the quality of the research studies, in general, studies adopted observational and retrospective methods with small sample sizes ranging from 20 semi-structured interviews [68] to a questionnaire with 700 respondents [64].

The six human rights investigation articles described violations in El Salvador during the civil war and the repressive regime that followed [35, 48, 59], in Nicaragua [56], Palestine (Gaza) [66] and South Sudan [44]. Four of the six were conducted or affiliated with human rights or advocacy organizations, one with Médecins Sans Frontières, and one with academic institutions. Methods for these papers included collection of witness testimonies and of physical and documentary evidence.

The six articles categorized as legal/policy analysis all conducted in-depth exploration of one or a handful of cases. Finally, five papers reviewed literature on attacks on healthcare since 2010. One study each focused solely on Syria and the Arab Spring, one on impacts of attacks on healthcare, and two on attacks on healthcare more generally. All reviews used scoping and qualitative methodologies and highlighted the lack of available and quality data. Information regarding the types, contexts, thematic domains and approaches of the articles are presented in Table 1.

#### Source of data analyzed in the literature

Reporting on incidents of attacks on healthcare in the literature was retrospective and has taken the following forms: (1) field level monitoring and reporting on attacks, collated into incident reports; (2) open-source data collection based on social media, news media and other publicly available reports; (3) in-depth mixed-methods case studies of specific countries, conflicts or incidents using interviews, inspections and evidence collection; (4) post-hoc investigations; or (5) impact studies. Of the 34 studies that used data (research and human rights investigations, excluding the reviews and analysis papers), 13 conducted only secondary analysis of previously-collected data and 21 studies collected original data. Of these 21, three conducted interviews to augment secondary data and 14 studies conducted qualitative interviews as the primary data source. Only four studies independently collected quantitative data.

**Fig. 2:**
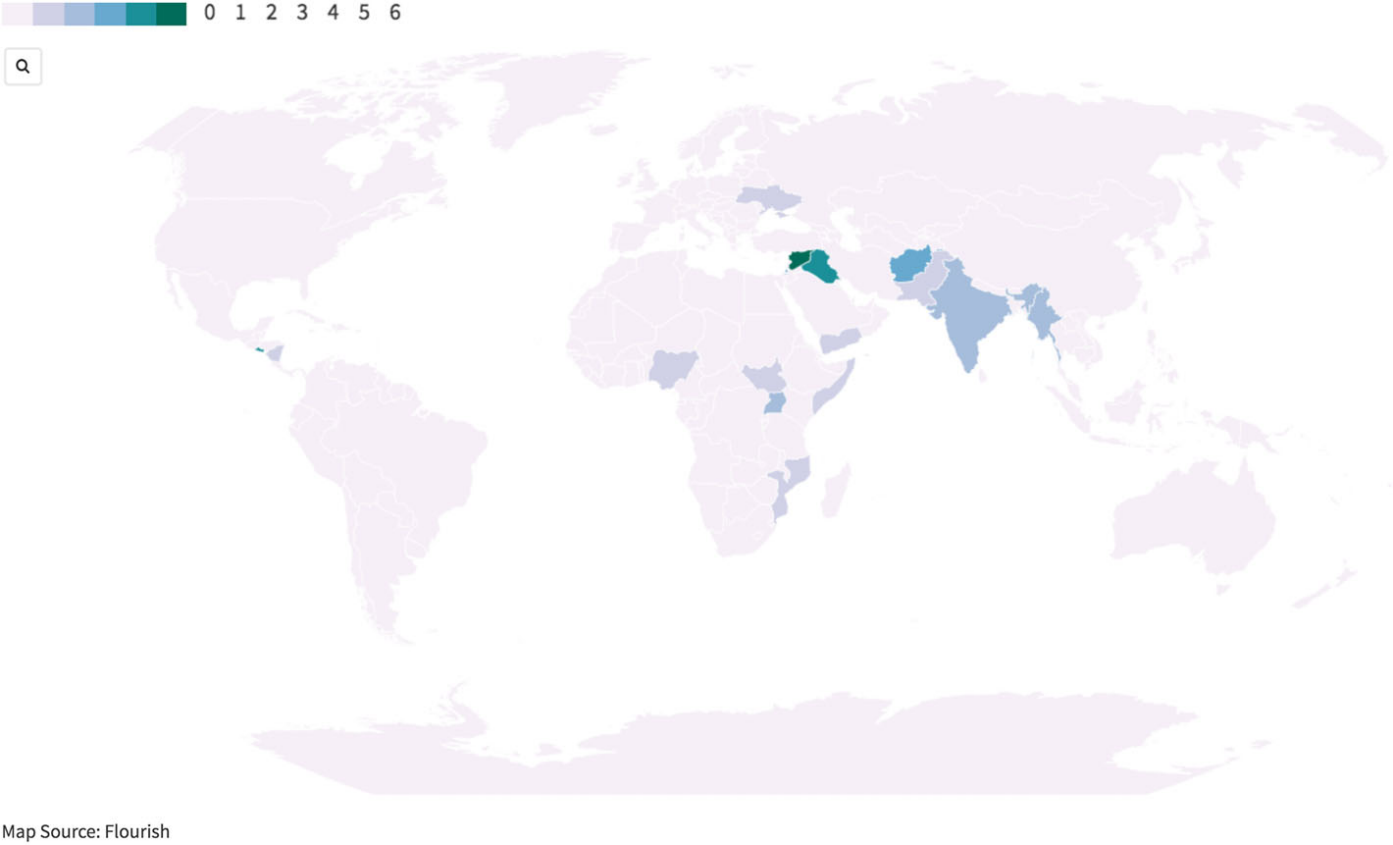
Number of studies by conflict/country

First authors were affiliated with 19 different countries: 19 were from the USA, three each from Canada and Switzerland, two each from Afghanistan, India, Lebanon, Sweden and the UK, and one each from Australia, Belgium, Denmark, France, Iraq, Israel, Mozambique, Norway, Pakistan and Turkey (Fig. 2). For authors with multiple affiliations, we scored the affiliation from the country with the highest ranking on the Fragile States Index [88].

### Thematic analysis of the literature

Analysis of the reviewed literature includes synthesis of (1) the state of available data and (2) overarching themes. The state of the data section includes results on the geopolitical focus, temporal characteristics, terminology used, characteristics of documented attacks, and methodology. The overarching themes, inductively defined, include the impacts of the attacks and lessons on mitigation and resiliency.

#### State of the available data

##### Geopolitical focus

Ten articles had no specific geopolitical focus. Of the remaining 35, one had a regional focus on Arabic speaking countries, while the other 34 focused on a single country and/or conflict (see Fig. 2). Several articles discuss sentinel events that could alter the nature of the discourse on attacks on health. For instance, the bombing of the MSF hospital in Kunduz, Afghanistan in October 2015, which brought widespread international attention to IHL and the culpability of the US military, is explicitly studied in four papers [34, 62, 80, 82]. Similarly, six papers focus on attacks on health in Syria [24, 49, 53, 60, 75, 85]. Other conflicts characterized by frequent attacks on health have been less well studied in the literature (i.e. four studies in Iraq [39, 40, 64, 68], three studies in Palestine [66, 77, 79], two studies in Myanmar [29, 53], 1 study each in South Sudan [44] and Yemen [70]).

#### Temporal characteristics

The attacks on health reported in these articles range from archival studies of historical conflicts [67] through the ongoing wars in Syria (2011-present) and Yemen (2014-present). Although attacks on health in conflict are not a recent phenomenon, the vast majority of the studies (40) were published between 2010 and 2019 (89%), and 2019 had the highest frequency of published studies (10), suggesting a growing interest in this problem.

#### Terminology

While ‘attack on health in conflict’ is the overarching framework as previously defined in the methods section, the terms “attack”, “health” and “conflict” are often undefined in the studies and, where they are, exhibit some variability in operational definitions.

##### Conflict

Eighteen articles mention the legal landscape, using the Geneva Conventions’ approach to considering when violence reaches the threshold of an international or non-international armed conflict. Twenty-seven articles have no explicit conflict definition. Some articles [43, 46, 69, 78, 83] document attacks on health in contexts of violence or political volatility that may fall below the threshold of an armed conflict under IHL. For example, one study in Nicaragua [83], which was in the throes of the political crisis in the late 1980s, documented violence by police and paramilitary forces in protests that resulted in extra-judicial killing, disappearances and detentions of health workers.

##### Attack

Defining an ‘attack’ was often imprecise. Although 25 of 45 papers explicitly define the characteristics of attacks, there is significant heterogeneity in the types of attacks studied. These attacks range from airstrikes to delays at checkpoints, threats and harassment of health staff, kidnapping and violent deaths.

Nine of 45 articles [39–42, 48, 58, 66, 68, 69] addressed impacts of conflict, human rights violations and indiscriminate violence on health professionals and services. The papers examined issues such as the impact of repression of women, including healthcare workers, by ISIS leaders on women’s ability to function freely [68], the effects of limitations on distribution of medical supplies and food [66], or how looting and pillaging of medicines and other resources can restrict health resources [41]. The literature contains examples of the impacts of chemical attacks on patients and health workers [53] and of delays of ambulances at checkpoints [66]. Many papers did not disentangle general disrespect for civilian lives and humanitarian law from attacks specifically on the health sector.

#### Research focus and findings

##### Attacks on personnel

Twenty of the 45 articles focused on documenting or analyzing attacks against health personnel, including doctors, nurses, and other clinical staff; an additional nine articles discussed personnel without focusing exclusively on them. Attacks on personnel include beatings and shootings [44, 50], as well as surveillance at work [68], arrest, intimidation or threats [29], obstruction of daily operations [48], or interference with obligations of impartial care [34]. While attacks on health workers occur in most places irrespective of ethnicity, a few articles point out how the experiences of health workers differ from those of the communities in which they work (‘local’), particularly when they are not from the specific geographic location or identity group. Those who are not ‘local’ may be at more risk of isolation from the host community and more vulnerable to attacks. In Burundi, attacks on health workers occurred ‘across ethnic lines’, and health workers “preferred to [work] in areas where they felt their safety and security was guaranteed, and that might be within their own community” [41]. Ethnicity or locality was found to be a factor in health worker vulnerability in the conflict in Myanmar as well [55], where ethnic groups fought the national government and the health workers provided care to targeted ethnic groups. There were anecdotal reports of similar vulnerabilities in South Sudan [44].

The gender dynamics of attacks on healthcare are not well-studied in general. Foghammar et al. [87] highlighted a knowledge gap around the ways that gender impacts on both the location and nature of attacks that health workers face. They observed that gender data may not be recorded or disclosed due to privacy concerns, or a lack of awareness of the importance of gender-sensitive data collection. One study of healthcare in Mosul under ISIS occupation noted that women were at risk of being forced to marry and not being allowed to travel or relocate, a practice also that also affected female health workers in particular [68]. Only one study focused solely on female health workers and considered the specific vulnerabilities of female community health workers in Pakistan [83]. It found that female community health workers were more likely to work in isolation in remote areas, with a risk of being targeted while doing preventative visits or vaccination campaigns. Additionally, attacks against them receive less attention and get reported less frequently. Only five papers [49, 52, 74, 83, 87] explicitly mentioned sexual violence as a form of attack that health workers may face, though three of these only noted that “it is very difficult to collect reliable data on sexual violence against health workers” because of stigma, and further violence from perpetrators, relatives and colleagues [87] and one remarked that no reports of sexual violence were recorded in their data collection [49]. None of the articles we included reported incidents or instances of sexual violence in either quantitative or qualitative data, though the paper on female health workers recorded threats of sexual violence [83].

##### Attacks on facilities

Attacks on facilities were the most commonly cited form of attack on healthcare in the papers reviewed. Facilities include clinics, hospitals, private offices and secondary health posts such as blood donation centers or medical education facilities. High profile hospital bombings in Syria, Yemen and Afghanistan have dominated the studies of attacks on facilities but incidents in other settings have highlighted the more insidious and chronic attacks [41]. For instance, a study in Mozambique highlighted that “196 peripheral health posts and health centers had been destroyed and another 288 had been looted and/or forced to close,” noting that this loss of health services has “hit people hardest in the rural areas where people are most in need of health care” [43]. In eastern Myanmar, qualitative interviews with health workers identified frequent attacks on clinics, which could not reopen because the military set up landmines around them [55]. Studies repeatedly noted the specificity of context and conflict. A study of hospital attacks in South Sudan in [44] illustrated variability in the nature of attacks that occur in different contexts within the same country, highlighting the importance of understanding conflict dynamics, historical context and perpetrators.

##### Attacks on transports

Only three papers specifically focused on attacks against ambulances and other medical transports [46, 77, 85] while 22 others included some information about attacks on transports. Chen and Wong used secondary analysis of literature to identify and characterize violent attacks on ambulances in Syria in 2016 and 2017 [85]. In other studies, delays at checkpoints, violence against mobile clinics and other transport-related violence featured more prominently than physical violence against ambulances [46, 55, 66, 70, 77, 83]. Incidents such as looting of medical equipment [41, 44], and disruption of medication cold chains [80] received little attention in the research literature on attacks on healthcare.

In a study of ambulances in Kashmir, the authors noted that police and paramilitary forces ‘did not allow them to use either the lights or the sirens. In fact, several drivers reported that they had been physically attacked by paramilitary forces for using a siren.’ [46]. Delays at checkpoints to inspect vehicles for weapons, refusal to allow ambulances to cross a thoroughfare and harassment and rerouting have most notably been documented in Palestine [66, 77, 79]. A study in Burundi and Northern Uganda identified few available ambulances and additional risks for travel at night, when ‘harassment, assault, or extortion by armed persons at roadblocks a common phenomenon” [41]. In Myanmar, two studies noted that destruction of mobile medical clinics, confiscation of supplies from mobile health workers and harassment of mobile health workers or patients while traveling. This limited their mobility in terms of the times of day they could travel and how much they could carry [29, 55]. A health worker in Myanmar reported that “*[the patient] should have traveled immediately to another clinic, but because of government soldiers we had to stay and cross late at night, so the patient was very severely ill when we arrived and we had to give IV treatment. Because of the delay he was far sicker on arrival*” [55]. In Syria, interviews in the Ghouta region suggested that women would schedule daytime C-sections to avoid the risk of going into labor and requiring transport to hospitals overnight [42].

##### Attacks on the wounded and sick

No papers focused exclusively on documenting attacks against patients but several described the experience of and impacts on patients secondary to a variety of attacks. Chukwuma et al., for instance, studied the effect of the Boko Haram insurgency on maternal health care utilization [42] and Trelles et al. [82] measured the averted health burden at the Kunduz trauma hospital. Other papers address attacks on patients secondary to the main domain of attack, and include data on killing and injury of patients, harassment and intimidation, blocking or interfering with timely access to care, denial of medical assistance and discrimination, and interruption of medical care through disruptions to medical functions [22, 24, 29, 41, 42, 44, 49, 55, 57, 58, 60, 66, 77, 82, 87]. Several articles pointed to the challenges of identifying patients as victims of health attacks, especially if not yet treated, when they are confused with bystanders and other civilians [29, 69, 77].

##### Misuse of health facilities and ambulances for military purposes

Attacks on health may target not only physical structures and persons, but may also misuse the symbols and the protected status of healthcare and/or undermine the integrity healthcare, primarily through the deliberate misuse of health for military or other purposes, in violation of the Geneva Conventions. Several articles cite the US military’s ruse of a fake vaccination campaign in Pakistan to further intelligence gathering [83], the use of ambulances to carry militants across checkpoints [79] and hiding weapons inside a health facility [80].

##### Perpetrators and intent

Three articles [37, 44, 67] focus on identifying the perpetrator or considering the motivations of perpetrators. Many others generally comment on perpetrators of specific attack, battle, or conflict. In most studies, perpetrators were identified as either State or non-State armed groups but several articles noted that disentangling conflict-related violence from interpersonal violence is challenging in practice. Studies in Iraq [64, 68] and Yemen [70] noted that interpersonal violence, including assaults on health workers by patients or families, did not disappear in conflict settings and may in reality reflect conditions of chronically insecure and underfunded health systems. They also noted that endemic violence may become more common in conflict settings where violence is more normalized in general [64, 68, 70]. An in-depth review of violence in South Sudan noted layers of complexity in circumstances of active conflict, particularly looting. Regarding an attack on a hospital in the town of Leer in 2014, the authors wrote, “Looting of the facility reportedly began in the last days when the staff were present and working, involving civilians and combatants alike in the panic and confusion created by the government’s offensive, including shelling the town. As one local witness recalled: ‘Light things like mats, medicines, items which can easily be picked up were taken by people from the community. Heavy machines were taken by the soldiers, both the rebels and the government soldiers and those who had joined the government” [44].

Identifying intent, or whether an attack was targeted or indiscriminate, is even more complicated than identifying perpetrators. As a result, intentionality is rarely addressed. The few discussions of intent focus on the challenges of understanding the motivations of attackers and note the complexities of any decision-making process [41, 67]. Crombé and Kruper wrote that “the joint product of interactions between rival political elites, and between these elites and local groups, down to individuals with their own interests, violence defies the maximization logics of any given set of actors” [44].

##### Overarching themes from the literature review

Several cross-cutting themes threaded through published research papers. These included: (1) vulnerabilities to attack, particularly related to healthcare visibility and emblems, (2) efforts at mitigating attacks or their effects and (3) attempts to study the consequences of attacks on healthcare.

#### Do the emblem or other markers of the medical profession heighten vulnerability?

At least four articles discussed how emblems of healthcare can either protect from or expose health services to violence [24, 60, 67, 80]. These emblems, most commonly the Red Cross or Red Crescent, are intended to designate protected services [67]. Studies in El Salvador, Iraq, Uganda, Afghanistan, Myanmar, Palestine, Syria, Yemen, Pakistan, and in the former Yugoslavia describe how the emblem has become a target and discuss strategies to avoid its use as a means of protection [24, 50, 55, 64, 67, 69, 79]. In South Sudan, signs of being a health worker, such as a white coat or possessing medical equipment, had to be hidden when security forces were present [44]. In Pakistan, emblems and identification as a health worker constituted a direct risk, particularly in remote areas in Swat Province [83]. In Kashmir, marked ambulances were at particularly high risk for targeted attack [46]. A study in Syria, where hospitals were systematically targeted, noted that only 14% of facilities and 31% of ambulances reported displaying a medical emblem in opposition-controlled Syria in 2016, and that “no significant difference (*p* = .208) in repeated attacks or probability of closure between hospitals with and without emblems in their limited dataset suggesting either that hospitals actively avoided being labeled and that the emblem did not protect from attack [60].

#### Prevention and mitigation

Many articles discussed prevention and mitigation efforts and included practical recommendations [24, 34, 44, 46, 53, 64, 67–70, 74, 79].

##### Protection and prevention

i) *Concealment,* in coordination with the local community, was discussed in several articles across continents. In Burma, Karen health workers hid in the forest and came out only when soldiers were asleep or moved away [55]. In South Sudan, health workers concealed their identify by removing medical paraphernalia and sleeping in the hospital among the patients, or frequently changing sleeping locations [44]. In Syria, hospitals removed medical insignia and moved to unmarked basements and caves to avoid being attacked [24, 50, 53]. Several articles note that concealment, however, does not mitigate risk from airstrikes, especially indiscriminate attacks [80].
ii) *Staff positionality and nationality.* Use of expatriate staff was discussed both as protection and vulnerability. Attacks on expatriates are more likely to cause international outrage. On the other hand, international staff may raise the profile of a particular health facility or service, making it more visible for attack or more attractive for hostage takers. In some instances, the presence of international staff (and their higher standards of living) may cause resentment among the local population, leaving them less allied with the local community and therefore less protected [46, 51, 52].
iii) *Negotiating security.* Literature on humanitarian negotiations exists but is beyond the scope of this review. Several papers, however, noted that negotiating directly with conflict parties and creating meaningful rapport was critical to protection. MSF had made significant progress in negotiating its neutrality with the Taliban and local government forces that may have protected from potential attacks ([34, 76] though the protection is incomplete and staff continue to be targeted. In Colombia, the Red Cross directly communicated with guerilla forces and invoked their obligation to care for fighters on any side [80]. Terry noted that the ICRC’s neutral stance makes denunciation of perpetrators’ violence more challenging [80]. In El Salvador, negotiations produced myriad trades: providing medical assistance in exchange for free passage at a checkpoint and to “less deserving populations in return for permission to provide services to needier ones” and in negotiating aid for policy concessions with governments [35].

##### Mitigation of impacts

i) *Task-shifting.* Several articles mention task-shifting as a way of limiting the effects of lost health workers or lack of adequate training. Community health workers, cut off from the broader health care system, were trained in El Salvador to be primary healthcare workers and treat basic conditions [35]. In Uganda, nurses learned to perform C-sections and physicians shifted across specialties to the field of most urgent need [41, 69].
ii) *Technology.* Some articles suggested that using remote technology, such as surgeries with advice from abroad (telemedicine), may be helpful in managing complex illnesses when local staff are not available [24]. However, these strategies have limitations: high-tech communication is often unstable in conflict settings, and without strong security precautions, may identify the health workers’ locations or contacts to hackers and attackers. ‘Low’ technology solutions may also serve as a mitigating factor: some studies noted that local health workers might be able to utilize natural remedies or make homemade cleaning supplies or feeding solutions more efficiently [35], increasing the flexibility and cost-efficiency of limited health services in conflict settings.
iii) *Protecting from physical damage*. Applying tempering film to the glass of buildings or ambulances to avoid glass shards, building hospitals inside bunkers and fortifying hospital (using sandbags, etc.) have been reported in various contexts to mitigate damage [80].

#### Impact of attacks

Fourteen of the papers touch on the wider, indirect, cumulative or long-term impacts of attacks on healthcare [22, 39–43, 48, 56, 57, 62, 68, 69, 77, 79]. There are numerous dimensions of impact – including personal impact on health workers (death, injury, attrition, emotional distress), patients (death, injury, intimidation from seeking care, poor health outcomes), as well on facilities and the health system or the general population.

The studies have primarily used qualitative methods to assess impact. Two papers utilized heath worker interviews and surveys to understand the root causes of attrition, by interviewing refugee doctors in Jordan [40], and the experiences of health workers under ISIS, by interviewing 20 doctors in Mosul who lived and worked under the ISIS regime [68]. A third study reviewed personnel records to understand why doctors left tertiary care hospitals in Iraq between 2004 and 2007) [39]. Through participant interviews and observations in Palestine, Sousa and Hagopian found that, in addition to other sources of interference, checkpoints, road re-routing and regular sieges “were clearly a barrier to delivering critical, acute care” [79]. Namakula and Witter used a life-history approach to understand the longer-term experiences of health workers in northern Uganda, finding both a sense of disconnection as well as loss of morale [69].

Quantitative studies included two from Afghanistan that assessed the impacts of the aerial attack on the Kunduz trauma hospital by describing patient characteristics and services utilized before and after the bombing to calculate the untreated burden of disease [62, 82]. In Palestine, Rytter et al. found that patients who experienced conflict-related delays (i.e. at checkpoints) were significantly more likely to be admitted to the hospital (32% vs. 13%, *p* < 0001) [77]. A study of rebel-led attacks in the 1980s in Mozambique used national level registry data to assess how the attacks were used to destabilize the health system and weaken the perception that Mozambique was able to self-govern effectively [43].

## Discussion

This review illustrates a diversity of approaches to researching attacks on healthcare with the aim of understanding how the research is harnessed for advocacy, protection and accountability efforts. Much of the academic research on violence against healthcare is qualitative and focuses on analyses of secondary data (either surveillance data or medical charting, in 16 of 34 articles). Only four research papers (11%) collected new quantitative data, underscoring the challenges of data collection in conflict settings. Qualitative studies have primarily elucidated the experiences of health workers and illustrated the range of violent and non-violent attacks health workers have suffered as well as the impacts on their personal and professional lives. The studies use open-ended interviews, surveys, life-history, historical case study and participant observation methods. Quantitative studies, both secondary analysis and original data collection, have primarily focused on documenting attacks, their features, scope and scale with a range of sources including self-reports, the use of data collectors, online sources, and other methods. Quantitative research has also been employed to study the health systems level effects of attacks or to compare these attacks across conflict incidents and time. The research describes attack trends, links publicly available attack data to health indicators, surveys health worker experiences and compares hospital records to conflict data to identify trends. These data provide insights on what is known in select countries at a moment in time but represents a small sample of violence against healthcare in conflict.

### When and where: *When and where are attacks happening or being researched?*

In nearly four decades since the first papers in this review (1983), there have been studies on 18 countries or conflicts. Over this period, the focus of research has shifted alongside global attention, from conflicts in Nicaragua in the 1980s, El Salvador and Yugoslavia in the 1990s, Iraq, Palestine and Kashmir in the 2000s, and Somalia, Syria, Nigeria, Uganda, Pakistan, Myanmar and Afghanistan in the 2010s. In the last decade, Syria has been the subject of 7 papers. However, many conflicts where violence against healthcare is significant and especially in Africa (the Democratic Republic of Congo, the Central African Republic, Mali, Sudan, and Libya), have not been subject to a single study.

Even within a country, the full geographic scope of the conflict is often not studied. For instance, attacks on healthcare have occurred for decades in Afghanistan but the three studies of attacks on healthcare in the country focused solely on the bombing of the Kunduz trauma hospital in 2015, likely because of global attention and U.S. responsibility. Even in the countries studied, only in Syria have there been assessments of methods of surveillance of attacks on healthcare.

As a result of the few studies conducted, and the absence of baseline data, no conclusions can be drawn on whether attacks on health are increasing over time. In sum, the research does not accurately portray where or when attacks on health are occurring but may give a perspective on the range and characteristics of attacks.

### What: *What types of attacks are being documented?*

There is a concern that high-profile airstrikes targeting healthcare facilities, and the advocacy around them, could skew attention away from other conflicts or other attacks on civilians, or misrepresent the scale of attacks globally [86]. However, this systematic review finds that many articles study less dramatic attacks including threats and interferences with healthcare. Myriad types of health facility and transport attacks are cited in the literature, including studies in Uganda, Somalia, Myanmar, South Sudan, DRC, Nicaragua, Palestine and region-specific studies in Kashmir and the Swat valley. The attacks include pillaging, looting, occupation, confiscating supplies, blocking entrance, or checkpoint delays. Among attacks on health workers, the vast majority studied in the literature are committed against local or national health workers and include threats, beatings, arrests, restrictions on work, torture and killing. Among these health workers, most studies focus on physicians and nurses but some cover community health workers, medics and other ancillary staff, depending on the context [41, 69, 83]. Gender dimensions of attacks on health workers and the specific risks for women, who may be more likely to work in rural areas or as community health workers, and may suffer from distinct forms of attack, are rarely addressed, as has been noted elsewhere [89]. The papers that specifically consider attacks on patients research killing and injury, harassment and intimidation, blocking care or interfering with timely access, denial of medical assistance, discrimination and interruption of medical care through disruptions to medical functions. The paucity of research overall suggests that much remains unknown about the global scale and types of attacks that occur, the objects of attacks, and their impacts.

### Why: *Why are attacks on health occurring?*

A key challenge in understanding attacks against healthcare is identifying perpetrators and their motivations [67]. In the studies in this review, circumstantial information such as the context, post-hoc legal evaluations and eyewitness accounts of surrounding events are used to attribute attacks. Rarely do these studies use direct evidence such as witness testimonies and self-identification [44, 70]. Except by implication, few studies addressed whether the violence was specifically targeted at heathcare, or whether it was an element of generalized or indiscriminate violence against civilians. In several papers, inferences about possible strategic reasons of the attack exist, such as to weaken the perception “of the government’s concern for the welfare of the people,” intimidate the population, or destroy hope of rebuilding in that area [43, 53]. As with other topics, much research remains undone in this realm.

### Addressing challenges

#### Towards clarity in the definitions

Synthesis of the literature has confirmed the need for more explicit discussion of the definitions and boundaries of this area of study. While some have suggested the need for consensus definitions to facilitate comparison across cases [22, 29, 49], the contexts of violence against health care are highly diverse and may require different approaches [41, 60, 86]. Instead, we argue researchers should endeavor to render explicit their definitions and discuss the implications of those decisions for their methodology.

### Conflict

The legal framework governing attacks on healthcare in conflict rests on the Geneva Conventions and its Additional Protocols, which apply during armed conflict. In some circumstances, international human rights law is more applicable [76]. However, some studies have looked beyond international and non-international armed conflict to address attacks within settings of political volatility and civil unrest [23, 56],where violence against medics, ambulances and wounded protesters mimics many of the conditions of conflict. Because the meaning of conflict is variable, referring to international, non-international, as well as politically volatile contexts, explicit delineation of the terminology of “conflict” is more important than consistency in definitions. However, in broadly defining conflict and potentially including civil unrest, it will remain crucial to continue to distinguish this range of conflict settings from interpersonal violence and attacks that occur in peacetime situations. In settings not within the IHL definition of conflict, human rights law as well as criminal law will still apply [54].

### Attack

Determining and classifying what counts as an attack on health in conflict was not always straightforward. Both violent and non-violent attacks constitute types of violence against healthcare. Non-violent interference included examples of intimidation, threats, and restrictions that profoundly harm health providers, patients and services. Beyond the attacks on facilities, transports, personnel and patients, misuse of health facilities and ambulances for military purposes, either violently or non-violently, also occurs frequently, and all may constitute violations of IHL. We conclude that it is critical to include physical and non-physical attacks, as well as direct and indirect attacks in any definition of violence against healthcare, as the impacts of the attacks are not always clearly correlated to the scale of the initial attack. While research should avoid equating airstrikes with threats of closure, for instance, both types of attacks can, in practice, halt service delivery for the local population.

Clarity is also needed to distinguish interpersonal violence in health care from conflict-related violence. Interpersonal violence persists in conflict and may even be exacerbated by the culture of aggression during armed conflict, but would not usually be understood as conflict-related violence per se. Furthermore, tensions among different communities and stress regarding sick family members, exacerbated by weak health systems with limited equipment and medical supplies (which may themselves be a result of attacks on health) may increase the frequency or scale of interpersonal attacks as respect for healthcare is eroded [40, 64]. Recent reports on attacks on health in the setting of COVID-19 only serve to heighten this concern [90, 91]. Moreover, it is difficult to distinguish interpersonal violence or criminal violence (e.g. robbery or gang violence), from politically-motivated violence. However, two critical factors in many papers distinguished interpersonal violence from the formal classification as “attacks on healthcare in conflict”: (1) the perpetration of violence against healthcare as politically motivated, whether specifically intended or as a result of indiscriminate violence and/or (2) when the perpetrator was an organized armed group or state actor. Attacks of this nature comprised the violence reported in these papers, which were framed around the obligations of ‘duty-bearers’ in armed conflict to ‘take precautions’ to protect civilian lives and medical care when engaging in hostilities.

#### Practical challenges to documenting attacks

Operationalizing any definition for those recording attacks adds another layer of complexity. Neuman addressed the potential differences between those doing the frontline documentation and those compiling data sets, and the organizational politics of recording insecurity:

‘At the project level for those whose responsibility it is to document incidents, there is no consensus on what exactly constitutes an incident. In a setting where violence and verbal threats are so prevalent, documenting insecurity represents a real challenge. Should the team only record events they consider to directly impact operations, such as shootings in the hospital, car jackings, etc., or should they try to document all incidents that occur, including minor threats, just to maintain a comprehensive record? The decision on whether to report an incident or not may be rooted in the fact that the person responsible for drafting the report wants to portray the reality in a specific light, whether that be to alert, alarm, or the opposite, reassure headquarters and the coordination team in Sanaa’ [70].

There are also challenges in characterizing or categorizing incidents. How do we account for multiple hits within a short, delineated timespan [49]? How do we account for attacks that span days or weeks such as the occupation of a facility or the kidnapping of a health worker [55]? How do we assign a classification of ‘healthcare attack’ for unclear situations? Additionally, naming protocols and language differences across contexts can make reporting consistency difficult. For instance, while post-British colonial countries may function under a centralized governorate➔ district➔subdistrict➔ community model, many countries, including the US, do not utilize this approach. Terms such as “medical point” or “mobile clinic” can imply different things. Even medical “transport” may refer to a fully functional ambulance in some settings and a motorcycle or boat in others. Health workers with the same titles may have vastly different responsibilities across settings [29]. In the US for instance, a receptionist may not have any clinical training and would not necessarily fall under an attack on a health worker while in Syria, a receptionist may be critical to clinical care, patient transport, vital sign measurement and other tasks. It is important, then, for researchers to consider not only how they understand and report attacks but also for stakeholders to explore the locally-held understandings of attacks and the victims. These challenges to standardization highlight a key challenge to producing globally aggregated data. Contextually specific, locally-held understandings of attacks may not be easily comparable. As with conflict definitions, we recommend clarity in definitions rather than a standardized attack definition which may miss these context-specific dynamics.

Fourteen of the articles address the indirect, cumulative or long-term impacts of attacks on health. There are numerous dimensions of impact – including those on health workers personally (attrition, emotional responses), patients (intimidation, health outcomes), as well on facilities and the health system as a whole or the population more broadly (population movements). None of the impact studies quantitatively link specific attacks to their impacts or probe the causal pathways in depth. There are also challenges in disentangling the impacts of attacks on health from the impacts of conflict and insecurity. Further research on the multiple wider, indirect and long term impacts of attacks on healthcare represents a promising opportunity to advance our understanding, and could draw upon impact studies in other fields.

### Limitations of this review

This study is limited, despite its attempt to synthesize a large body of literature. The papers that we identified were dispersed among a variety of disciplines and formats, which made using any single search inadequate. We started with a systematic search to collect as many articles as possible but our final articles for inclusion emerged with nearly half of the papers identified through reference review and professional networks, highlighting the challenges of systematic review for interdisciplinary issues. We did not include the large body of human rights and other reports, or data-collection initiatives (for example, the ICRC’s Healthcare in Danger Initiative) that have addressed the problem of violence against healthcare. We chose to exclude these reports in this review as current search strategies were not successful in identifying all the key grey literature, especially for older reports before the internet was in wide use. Additionally, though grey literature publications have many useful insights, they usually consist of documentation of attacks or summaries of data. Many do not describe methods used. For the same reason, we excluded commentaries and perspective essays, though they often included important points. Reviewing these reports and initiatives would add significantly to the lessons learned on attacks on health [76] but were not within the scope of this review focused on peer-reviewed research.

Although we attempted to be comprehensive, we may not have captured all the papers written on this subject. We acknowledge the positionality of the authors, who wrote some of the reviewed papers and have engaged members of the civil society community working to advocate against attacks on healthcare. This positionality has informed the analysis but we have worked to utilize the depth and range of our collective experience to bring greater clarity and nuance to this paper. We only searched the English-language literature but further scoping of the Spanish, Arabic, Farsi, French, Hindi and other languages might reveal far more research. While we attempted to be thorough in our search terms, there may be some terms that yielded information about attacks on health that we excluded. The articles were heterogenous and hard to categorize, therefore, perhaps a defined taxonomy and engagement of dispersed researchers interested in this topic would be useful for future work.

## Conclusions

This paper reviews 45 articles, of which 35 were research studies and 10 were analyses, over nearly 40 years to better understand how attacks on health in conflict are documented, studied and used for prevention, protection, and accountability. The studies provide insight into particular cases of violence against healthcare in armed conflict, but significant gaps exist regarding many contexts, characteristics of violence, and methods of documenting attacks and their implications. These underscore the need for future research. The studies offer lessons on the use of novel investigative methods as well as mitigation strategies in response to the violence. Cumulatively, these data underscore the context-specific nature of the purposes, means, and impacts of violence against healthcare. While there is much to be learned from collecting global data, measuring the scale of attacks, and standardizing reporting, the work must stay rooted in the context. Promisingly, the studies, written by physicians, researchers, legal experts, human rights investigators and others showcase the strengths of heterogenous and interdisciplinary approaches.

The literature has expanded in the past 10 years but it is unclear if the knowledge generated has helped to curb the frequency, scale or scope of incidents of attacks on health in conflict. Key questions remain: are states and armed groups modifying their behavior? Is there accountability for these crimes? The question of what actions are needed from states, armed groups, NGOs, UN agencies and civil society to prevent attacks is beyond the scope of this paper, but it is evident that further research, standardized monitoring and their use for reform, programming and accountability are critical. Further, stakeholders must ensure the relevance of research toward these ends. We also need a better understanding of the consequences of the attacks both in the short and long terms and on local, national and international impacts [92]. There is much at stake in ensuring we understand attacks on healthcare and this review has highlighted some of the key avenues for future research and policy action (Table 2).

**Table 2:** Potential Future Steps in Studying Attacks on Health in Conflict

*Agenda on Improving Data Collection and Utilization*	• Explicit definitions and descriptions of key terms, such as conflict and attacks would aid in better understanding of the topic and allow for more comparisons across professions and conflicts. • The ‘misuse of health facilities and ambulances for military purposes’ category will be critical to include on ongoing data collection mechanisms, as it allows for documenting significant attacks that are not well-classified into the other discrete categories. • Given the redundancy in data collection, with multiple actors collecting and collating data in the same areas, there must be more availability of and accessibility to disaggregated data (with all the security precautions) to make this work more efficient. • Continued research on attacks on health and on civilian protection in conflict must become a bigger funding priority.
*Agenda on Deepening Research*	• Exploration of gender dynamics in attacks on health, both in terms of the gendered nature of forms of attack and the broader gendered impacts that attacks may generate, will be a critical next step. • Disentangling the experiences and perceptions of expatriate, national and local health workers, their interactions with each other and with the community, especially in settings of insecurity and where they may work together closely but have different experiences and responses. It is especially important to consider the overlapping and intersectional nature of these identities and to distinguish the specific vulnerabilities they raise (e.g., the differences, between a local health worker working in their national health service and a local health worker working for an international NGO). • Legal analysis of the weaknesses of IHL, such as data on the number of prosecutions and their outcomes, potentially linked to a better understanding of other IHL protections if they exist. Legal analysis could also continue to explore other accountability mechanisms, such as the United Nations Security Council, the ICC, ICJ and local, national or regional courts. • There must be more in-depth analysis and discussion of chronic and/or small-scale attacks as there is early evidence that these can be insidious and have reverberating impacts on the community and health workers. • The geographic focus of research needs to continue to be expanded, as attacks are known to be global and pervasive. • Studies looking at the cumulative impacts of attacks, including behavioral changes, population movements, or health outcomes would expand our knowledge of the effects of attacks. • More research is needed on perpetrators and intentionality, especially on the dynamics in specific contexts and conflicts. With better understanding of why specific attacks happen in specific conflicts it will be possible to design more effective protection strategies and interventions designed to elicit behavior change in perpetrators. • Research which analyses the processes of collecting data on attacks on health and how it may be used operationally among humanitarian organizations to protect from future attacks would provide valuable insights.
Agenda on achieving meaningful change:	• Utilizing ICC, ICJ and national frameworks for legal accountability in addition to traditional IHL frameworks. • It goes without saying that perpetrators need to curb their attacks on health. More research into perpetrator motives and the operationalization of insights from research into what influences the behavior of armed groups is necessary for this [93, 94]. • Allocating resources to disseminate research to local and national actors. • Involving and engaging survivors and local actors in all levels of the research and dissemination.

In recent years there have been a number of initiatives apart from the research reviewed here to expand data collection on violence against healthcare, though many are under-resourced [6, 21, 95]. The WHO is beginning to take on a role in collecting and disseminating limited aggregated information in some countries based on a mandate from the World Health Assembly in 2012, but it is subject to political pressures and bureaucratic hurdles [5, 96]. The WHO system has potential, though it needs to be methodologically strengthened, expanded in scope, better coordinated with country offices and external stakeholders, and have disaggregated and more detailed data available (including on details of an attack and on perpetrators) before it can become the global focal point for data on violence against healthcare. Further, neither the research nor data collection has resulted in state action to protect healthcare in conflict, as provided in UN Security Council Resolution 2286 (2016). Perpetrators of violations of IHL, including attacks on health, may be investigated but to date prosecutions have not been initiated. States and international organizations must make addressing attacks on healthcare a priority, in terms of supporting research, prevention and response. This, too, is unfinished business.

## Supplementary Information

**Additional file 1: Supplementary Table 1.** Search terms for systematic review.

**Additional file 2: Supplementary Table 2.** PRISMA Checklist.

## Data Availability

Not applicable.
