# Supplementary material for: Violence against healthcare in conflict: a systematic review of the literature and agenda for future research
Source: Confl Health. 2021 May 7;15:37. doi: 10.1186/s13031-021-00372-7 (PMC8103060; doi:10.1186/s13031-021-00372-7)
Supplement: Supplementary file 1 — Additional file 1: Supplementary Table 1. Search terms for systematic review. [file 13031_2021_372_MOESM1_ESM.docx]

**Supplementary Materials**

Supplementary Table 1: Search terms for systematic review

| Attack | Health | Conflict |
| --- | --- | --- |
| abuse | ambulance | armed |
| access | care | armed conflict |
| airstrike | clinic | conflict |
| arrest | doctor | emergency |
| assault | facility | fragile state |
| attack | health | humanitarian |
| beaten | health personnel | international armed conflict |
| block | health staff | international humanitarian law |
| bomb | health worker | non-international armed conflict |
| detain | healthcare | uprising |
| guard | hospital | war |
| interference | ill |  |
| interrogate | medical care |  |
| intimidate | medical clinic |  |
| kidnap | medical facility |  |
| kill | medical transport |  |
| loot | medicine |  |
| obstruction | nurse |  |
| occupy | patient |  |
| protect | physician |  |
| rape | public health |  |
| sexual violence | sick |  |
| shoot | surgeon |  |
| stabbed | transport |  |
| target | wounded |  |
| theft |  |  |
| threat |  |  |
| torture |  |  |
| violence |  |  |
